# An INS‐1 832/13 𝛽‐Cell Proteome Highlights the Rapid Regulation of Fatty Acid Biosynthesis in Glucose‐Stimulated Insulin Secretion

**DOI:** 10.1002/pmic.70005

**Published:** 2025-07-20

**Authors:** Nina Stremmel, Oliver Lemke, Kathrin Textoris‐Taube, Daniela Ludwig, Michael Mülleder, Julia Muenzner, Markus Ralser

**Affiliations:** ^1^ Institute of Biochemistry Charité – Universitätsmedizin Berlin Berlin Germany; ^2^ Core Facility High‐Throughput Mass Spectrometry Charité – Universitätsmedizin Berlin Berlin Germany; ^3^ The Centre for Human Genetics Nuffield Department of Medicine University of Oxford Oxford UK; ^4^ Max Planck Institute for Molecular Genetics Berlin Germany

**Keywords:** glucose‐stimulated insulin secretion, pancreatic beta cells, quantitative proteomics

## Abstract

Pancreatic beta cells secrete insulin in response to rising glucose levels, a process known as glucose‐stimulated insulin secretion (GSIS). Here, we acquire proteomes of rat pancreatic INS‐1 832/13 beta cells that were short‐term stimulated with 11 different glucose concentrations from 0 to 20 mM, quantifying the response of 3703 proteins. Ensemble clustering of proteome profiles revealed unique response patterns of proteins expressed by INS‐1 832/13 cells. Three hundred and fourteen proteins, amongst them proteins associated with vesicular SNARE interactions, protein export, and pancreatic secretion, increased in abundance upon glucose stimulation. In contrast, many proteins implicated in metabolic glucose sensing processes such as glycolysis, the TCA cycle, and the respiratory chain, did not respond. Interestingly, we observe that enzymes participating in fatty acid metabolism showed a “switch‐on” response upon release of complete glucose starvation with no further changes in abundance upon increasing glucose levels. We speculate that increased activity of fatty acid metabolic activity might either be part of GSIS by replenishing membrane lipids required for vesicle‐mediated exocytosis and/or by providing an electron sink to compensate for the increase in glucose catabolism. These findings offer new insights into beta cell function and may inform future strategies for targeting metabolic pathways in diabetes treatment.

**Summary:**

- We used high‐throughput proteomics to capture comprehensive proteome changes 30 min post stimulation in the INS‐1 832/13 beta cell line, a commonly used cell model in studying glucose‐induced insulin secretion.
- Our results show that specific parts of the proteome respond promptly upon glucose exposure in this cell line. Furthermore, while many proteins canonically associated with GSIS did not change in abundance in the time frame and cell line investigated, our results attribute a specific role to fatty acid biosynthesis in the early steps of insulin secretion.
- By documenting protein abundance alterations in the initial phase of GSIS in the INS‐1 832/13 beta cell line, our study highlights the necessity of sampling early time points, well‐controlled study design and biological replicates in the study of beta cell function.

## 1 Introduction

Pancreatic islets are assemblies of endocrine cells spread throughout the pancreas. Endocrine beta cells account for the majority of islet cells in all vertebrates and secrete insulin in response to rising blood glucose levels. Impaired insulin secretion causes Type‐1 and Type‐2 diabetes mellitus, which together affect around 589 million people worldwide [1]. Glucose‐stimulated insulin secretion (GSIS) is highly regulated [2, 3] and occurs in two phases. When plasma glucose levels increase after food ingestion, the uptake of glucose via glucose transporters increases simultaneously [4, 5]. The canonical secretion model describes that during the triggering phase—immediately after glucose uptake—glycolytic degradation of glucose to pyruvate results in enhanced oxidative phosphorylation. This gives rise to an increased intracellular ATP/ADP ratio and subsequently triggers a chain of K_ATP_ channel‐mediated depolarization events leading to exocytosis of insulin‐filled vesicles [6]. The following amplifying phase is K_ATP_ channel‐independent [7] and allows for reduced, yet steady insulin secretion throughout the post‐absorptive phase of a meal.

Despite being largely consistent with the electrochemical characterization of pancreatic beta cells, the canonical model does not comprehensively explain all aspects of GSIS. For example, the role of the respiratory chain as a key producer of ATP that closes K_ATP_ channels has been challenged recently [8, 9, 10]. Moreover, several additional pathways and coupling factors such as the pyruvate cycling pathways, the pentose monophosphate shunt pathway and its nucleotide products, and the role of the glycerolipid/FFA cycle have been suggested to contribute to GSIS [11, 12].

Transcriptomic, metabolomic, and phosphoproteomic approaches have been used to study the early response of pancreatic beta cells to glucose, with the latter two in particular offering insight into rapid stimulus‐induced changes [13, 14, 15, 16, 17]. Nonetheless, many cellular processes depend directly on the regulation of protein levels, making the proteome response a crucial factor in insulin secretion mechanisms. Previous proteomic work studied the effects of 24–72 h exposure to compounds that trigger insulin secretion [18, 19, 20, 21, 22]. However, protein level changes can act faster, for instance through the interplay of protein synthesis and degradation. We herein explore the proteomes of INS‐1 832/13 beta cells during a 30‐min exposure to varying levels of glucose, ranging from zero glucose up to very high glucose concentrations. We identify rapid and specific proteomic changes through an ensemble clustering approach, revealing 11 superclusters of proteins with similar concentration‐dependent responses. The most strongly changing clusters were enriched for proteins regularly attributed to GSIS, such as those functioning in vesicle transport and excretion. Furthermore, our analysis highlights the rapid regulation of fatty acid biosynthetic enzymes following GSIS in the INS‐1 832/13 beta‐cell model.

## 2 Methods

### 2.1 Reagents

**Table:** 

**Cell line**	**Vendor**	**Cat.‐No**.
INS‐1 832/13	Merck	SCC207
**Assay/Plates**	**Vendor**	**Cat.‐No**.
Rat Insulin ELISA	Mercodia	10‐1250‐01
BCA 660 nm assay	Pierce	22600
BioPureSPE Macro‐ 96‐well Proto 300 C18	The Nest Group	HNS S18V‐L
Quantitative fluorometric peptide assay	Pierce	23290
**Reagent**	**Vendor**	**Cat.‐No**.
RPMI 1640	Sigma–Aldrich	R0885
Fetal calf serum	Biochrom AG	S0115
HEPES	Gibco	15630‐056
Glutamine	Millipore	K0283‐BC
Sodium‐pyruvate	Millipore	TMS‐005‐C
Penicillin	Millipore	A2213
Streptomycin	Millipore	A2213
Beta‐mercaptoethanol	Thermo Fisher	21985023
Glucose‐free RPMI 1640 medium	Gibco	11879020
Glucose	Gibco	A2494001
Urea	Sigma–Aldrich	U5128
Ammonium bicarbonate	Carl–Roth	T871.2
Magnesium chloride	AppliChem	131396
Benzonase	Millipore	101654
Dithiothreitol	Sigma–Aldrich	43816
Iodoacetamide	Sigma–Aldrich	I1149
Trypsin Lys‐C	Promega	V5072
Formic acid	Fisher Scientific	A117‐50
Methanol	Fisher Scientific	10031094
Acetonitrile	Fisher Scientific	10489553

### 2.2 Cell Culture

INS‐1 832/13 cells were cultured in RPMI 1640 medium containing 11 mM glucose and supplemented with 10% fetal calf serum (FCS), 10 mM HEPES, 2 mM glutamine, 1 mM sodium‐pyruvate, 50 mg penicillin, 50 mg streptomycin, and 1× beta‐mercaptoethanol. Cells were incubated at 37°C in a humidified atmosphere at 5% CO_2_. The medium was exchanged every two days, three times a week. Cells were passaged weekly with a split ratio of 1:20.

### 2.3 Glucose Treatment and Sample Harvest

Cells were seeded in 12‐well plates at 0.5 * 10⁶ cells/well. After 20 h of incubation, cells were washed twice with 1 mL of glucose‐free RPMI 1640 medium supplemented with 10% FCS, 10 mM HEPES, 1 mM sodium pyruvate, 50 mg penicillin, 50 mg streptomycin, and 1x beta‐mercaptoethanol. Afterward, the medium was replaced with 1 mL of RPMI 1640 supplemented with 3 mM glucose and cells were incubated at 37°C, 5% CO_2_ overnight to equilibrate to the lower glucose concentration. After overnight pre‐incubation at 3 mM glucose, cells were washed twice with 1 mL glucose‐free medium before 1 mL of stimulation medium (RPMI 1640 supplemented with 0, 2, 4, 5, 6, 7, 9, 10, 12, 15, or 20 mM glucose) was added. After adding the stimulation medium, cells were incubated for 30 min. The supernatant of each well was centrifuged at 400 × *g*, 4°C for 5 min, and immediately assayed by ELISA in a 1:5 dilution following the manufacturer's instructions. The cells were carefully washed twice on‐plate with the respective stimulation medium before they were lysed on the plate using 100 µL of lysis buffer (8 M urea, 50 mM ammonium bicarbonate, 2 mM magnesium chloride, and freshly added 100 U benzonase) to extract proteins. Cell lysates were transferred to Eppendorf tubes and centrifuged at 16000 × *g*, 4°C for 10 min. Supernatants were stored at −80°C until further processing.

### 2.4 Proteomics Sample Preparation

The protein concentration of lysates was quantified using a commercial bicinchoninic acid (BCA) 660 nm assay according to the manufacturer's instructions. Fifty micrograms of protein were processed per sample (in 8 M urea, 0.1 M ammonium bicarbonate). To reduce disulfide bonds, 5 mM dithiothreitol (final concentration) was added to each sample, followed by incubation at 30°C for 1 h. Next, 5 µL of 0.1 M iodoacetamide were added to each sample to acetylate previously reduced thiol groups. After an incubation of 30 min at 20°C in the dark, samples were diluted by adding 340 µL of 0.1 M ABC before digestion with 1.5 µg of trypsin/LysC per sample. Samples were incubated overnight at 37°C. Digestion was stopped by addition of 20 µL of 20% formic acid (FA). Peptides were purified by solid‐phase extraction (SPE). SPE plates were activated by adding 200 µL/well of methanol. After centrifugation (50 × *g* for 1 min), 200 µL/well of 50% acetonitrile (ACN) were added, followed by another centrifugation step (150 × *g* for 1 min). The addition of ACN and the following centrifugation was repeated once. The flow‐through was discarded and 200 µL/well of 0.1% FA were added. The plate was then centrifuged again (150 × *g*, 1 min). This step was repeated once before the flow‐through was discarded and 400 µL/well of the digested sample were added in two steps. After centrifugation (150 × *g*, 1 min), 200 µL/well of 0.1% FA was added, followed by another centrifugation (150 × *g*, 1 min). The addition of FA was repeated three times. A collection plate was placed underneath the SPE plate and the samples were eluted by three steps of addition of 110 µL of 50% ACN followed by centrifugation (200 × *g*, 1 min). The eluted samples were then dried in a vacuum concentrator and subsequently resuspended in 60 µL of 0.1% FA. To determine the peptide concentrations of the samples, a quantitative fluorometric peptide assay was carried out following the manufacturer's protocol. Six process quality controls were prepared by pooling all samples, and then distributing this pooled sample once per row on the plate before peptide purification. Technical quality controls consisted of repeat injections of a single purified process quality control sample every 10 measurements, resulting in six technical controls.

### 2.5 LC–MS/MS Measurements

Peptide separation was performed on an Ultimate 3000 RSLnanoHPLC (ThermoFisher Scientific). Tryptic peptides (1.25 µg/sample) were loaded on a trap column (PepMap C18, 5 mm × 300 µm × 5 µm, 100 Ǻ, Thermo Fisher Scientific) and separated on an analytical LC column (Acclaim PepMap C18, 200 mm × 75 µm × 2 µm; 100 Å; Thermo Fisher Scientific) at a flow rate of 250 nL/min. The mobile phase consisted of 0.1% FA (buffer A) and 80% ACN, 0.1% FA (buffer B). Digestion products were resolved using a linear gradient (3%–20% buffer B in 80 min, followed by 20%–45% buffer B in 10 min and 98% buffer B in 1 min). Total acquisition time per sample was 115 min. The eluate was directed to a Q Exactive Plus Hybrid Quadrupole‐Orbitrap mass spectrometer (ThermoFisher Scientific), which operated in centroid mode. One MS1 scan was conducted at 35k resolving power with a maximum injection time of 60 ms, followed by 51 MS2 scans at 17.5k resolving power with a maximum injection time of 60 ms. The window length for the MS2 scans was set to a mass‐to‐charge ratio (*m*/*z*) of 24.0 using an overlapping window pattern. Samples were measured in data‐independent acquisition mode and analyzed with the software DIA‐NN [23]. Every tenth injection was a technical QC sample. Additionally, the six process QCs samples were randomly injected during the measurements.

### 2.6 Pre‐Processing of Proteomics Data

Raw proteomic data were processed using DIA‐NN 1.8 (https://github.com/vdemichev/DiaNN) [23] using the default settings with fragment ion *m*/*z* range set from 300 to 1800, mass accuracy set to 20 ppm at the MS2 and 10 ppm at the MS1 level, respectively, scan window set to 11, MBR (Match‐between‐runs) enabled, and the Quantification strategy set as “Robust LC (high Precision)”. To correctly assign bovine protein contaminations stemming from the use of FCS in the cultivation and wash medium, two FASTA files were provided for the generation of an in‐silico spectral library and gene annotation: a Bos taurus reference proteome (UniProt UP000009136, accessed second May 2022), and a Rattus Norvegicus reference proteome (UniProt UP000002494, accessed 15th September 2021). Species‐specific gene interference was enabled. The report.tsv file resulting from the DIA‐NN run was further processed at the precursor level using normalized precursor intensities. First, all non‐proteotypic precursors and precursors with PG.Q.Value, Global.Q.Value, Global.PG.Q.Value, GG.Q.Value > 0.01, and precursors exhibiting a CV > 30% across the technical QCs (repeat injection) were removed. At this step, one technical control (QC_002) was excluded since the distribution of the fold changes of all precursor abundances in technical control QC_002 compared to their respective mean value over all technical replicates was clearly shifted compared to the other technical controls (quality control filter suggested by [24]). Furthermore, samples that had too few or too many precursors after these filtering steps (Z‐score of precursor number < −1.5 or > 1.5) were excluded (six samples, two quality controls). Next, only genes quantified by ≥ 2 precursors, and genes quantified by exactly one precursor but detected in at least 75% of all replicates were retained. Based on this set of precursors, protein quantification was performed using the diann_maxlfq() function of the DIA‐NN R package (https://github.com/vdemichev/diann‐rpackage). Bovine proteins were filtered out for all further analyses. The dataset contained 7109 proteins before the above‐described filtering, of which 3703 proteins were quantified after filtering and included in further analyses.

### 2.7 Statistical Analysis

#### 2.7.1 Clustering Analysis

For the cluster analysis, an ensemble clustering approach was adapted as described in [25]. For the ensemble clustering, the spatial *kMeans(++)*‐ [26, 27], the density‐based *commonNN*‐ [28, 29, 30, 31], as well as the community‐detection *Leiden*‐algorithm [32] were combined. Each algorithm can capture a different property of the data set. The network‐based Leiden‐algorithm captures connectivity between the data points providing a coarse structure of the data set, while the other two algorithms account for more spatial properties. The kMeans(++)‐algorithm accounts for the distribution and spread of the data points creating sphere‐like clusters of similar size, while the commonNN‐algorithm accounts for the density distribution resulting in size‐ and shape‐independent clusters. Since the *kMeans*‐ as well as the *commonNN*‐algorithms are parameter‐dependent, different parameter sets were included. For the *kMeans*‐algorithms, the number of clusters *k* was varied between 10 and 49 in steps of 1 (40 different cluster results). For the *commonNN*‐algorithm, the number of nearest neighbors in common *N* was varied between 2 and 10 in steps of 1, the distance cutoff R between 0.7*R_cut and 1.1*R_cut in steps of 0.1*R_cut, where *R_cut* denotes the distance at which roughly 0.1% of all distances between the samples are smaller (45 different parameter combinations). The minimal number of samples per cluster *M* was fixed to 5. For the *Leiden* clustering, the seed was fixed to 42 and a directed 1‐scaled(distance)‐weighted network with *n_neighbors* = 10 was used, utilizing the *ModularityVertexPartition* algorithm. For the distance estimations, we used the Euclidean distance as it is on the one hand easy to interpret and on the other hand adds a high penalty to points more distant in at least one dimension.

For each algorithm, a separate co‐clustering matrix was constructed and normalized by the number of different cluster results, where each element accounts for the probability that two samples were clustered together. The three co‐clustering matrices were combined with equal weights and clustered again using hierarchical Ward‐clustering [33] to detect proteins that are frequently clustered together. The clusters were extracted using a dynamic linkage‐based cutoff. Due to the used algorithms, a block‐diagonal matrix was obtained which was mainly driven by the Leiden‐clustering, whereas the other two algorithms provided the fine‐structure. In the end, 11 superclusters with in total 104 clusters were extracted. As the experiment follows a glucose gradient, the dimensions can be interpreted as concentration‐dependent changes in protein quantity. Thus, the distance between two data points denotes the profile similarity between two proteins. Due to this, the resulting superclusters and their clusters contain proteins sharing a similar glucose concentration‐dependent profile with similar amplitudes. The analysis was carried out using *Python 3.9.13*, *numpy 1.22.4* [34], *scikit‐learn 1.1.1* [35], *igraph 0.9.9* [36], *leidenalg 0.8.9*, and *scipy 1.8.1* [37]. The used functions are available under https://github.com/OliverLemke/ensemble_clustering.

Statistical significance of variance between conditions within clusters was calculated using the one‐way ANOVA test. Multiple hypothesis correction to adjust *p* values was applied (Benjamini–Hochberg correction) [38]. Since we tested 10 conditions per cluster and ANOVA shows to be significant as soon as one condition is significantly changed compared to the condition 2 mM, the majority of clusters showed significant changes with a cut off at *p* values < 0.05. Therefore, adjusted *p* values were considered significant when *p* value < 0.0001.

#### 2.7.2 Over‐Representation Analysis (ORA)

ORA was carried out using WebGestalt 2019 [39] using default settings on KEGG pathway terms [40]. A list of all 3703 consistently quantified proteins was used as the reference gene list and results were filtered at a false discovery rate (FDR) < 0.05. Multiple hypothesis correction to adjust *p* values was applied (Benjamini–Hochberg correction) [38].

#### 2.7.3 Protein Network Analysis

Protein network analysis was carried out using the search tool for the retrieval of interacting genes (STRING, version 12.0) using default settings [41]. A list of the 21 proteins that were identified to drive the ORA was used. The minimum required interaction score between proteins was set to 0.4. The network edges were connected by confidence.

#### 2.7.4 Differential Expression Analysis

Differential expression analysis was carried out using the *limma* package in R [42]. Linear models were fitted by the *lmFit()* function. Subsequently, empirical Bayes moderation was applied using the *eBayes()* function, resulting in log_2_FC and associated *p* values. Multiple hypothesis correction to adjust *p* values was applied (Benjamini–Hochberg correction) [38].

## 3 Results

### 3.1 The INS‐1 832/13 Beta Cell Proteome After Short‐Term Glucose Exposure

**FIGURE 1:**
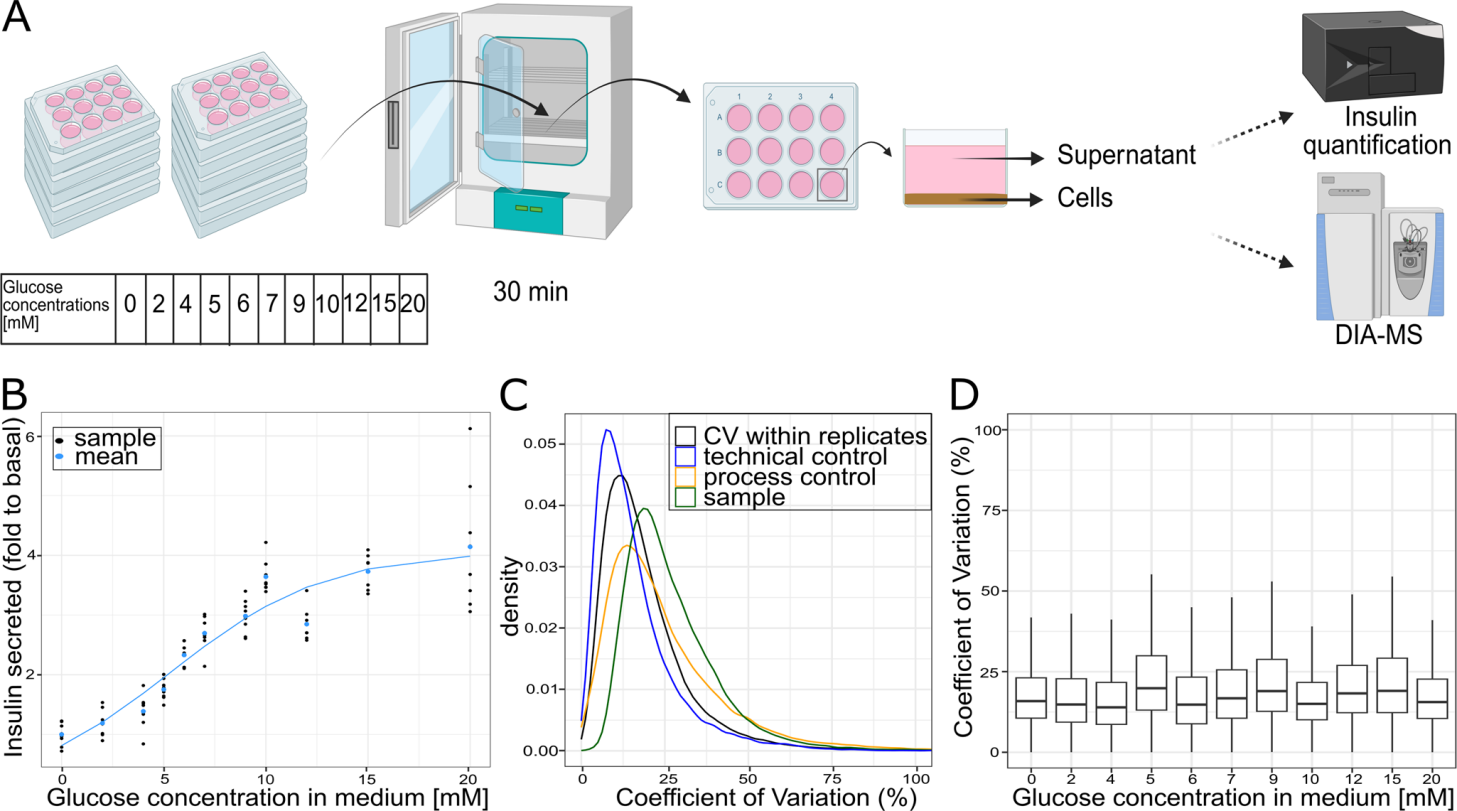
(A) Experimental design of the glucose stimulation dose‐response assay. INS‐1 832/13 cells were cultivated in 12‐well plates. Eleven glucose concentrations between 0 and 20 mM were used to stimulate cells for 30 min at 37°C, before the supernatant of all samples was used for insulin quantification by an enzyme‐linked immunosorbent assay (ELISA), and the cells were prepared for proteomic analysis. (B) Glucose concentration‐dependent insulin secretion of INS‐1 832/13 cells shown as fold change to basal (0 mM glucose) secretion. Individual replicates (*n* = 8) are shown in black dots and the mean of all replicates per condition is shown in blue dots. The data is fitted by a logistic function (blue line), with a residual standard error of 0.48 on 85 degrees of freedom. (C) Comparison of coefficients of variation (CV) based on precursors within experimental groups (black), across all biological samples (green), within repeated technical control injections (blue), and sample preparation process controls (orange). (D) Comparison of CVs based on precursors within replicates, separated by conditions. The median is represented by the line inside the box. The box itself represents the interquartile range between the 25th and 75th percentile. The whiskers indicate the range within the 1.5 * interquartile range.

To characterize the dynamics of the beta cell proteome of the INS‐1 832/13 beta cell line at an early time point of GSIS, we recorded glucose concentration‐dependent insulin secretion and changes in protein abundances after glucose stimulation of 30 min in the pancreatic beta cell line INS‐1 832/13 (Figure 1a). Cells were stimulated with eleven different glucose concentrations between 0 and 20 mM, covering complete glucose starvation (0 mM), the physiological resting blood glucose level (3–5 mM), postprandial blood glucose levels (7–8 mM), as well as high glucose levels often used in long‐term glucose exposure studies (15–20 mM) [43, 44, 45, 46]. In order to render our results comparable to existing metabolomics data, the glucose concentrations in the different conditions were chosen to align with a previous metabolomic study in the same cell line [17]. Prior to stimulation, cells were incubated overnight at 3 mM glucose to adapt to resting glucose levels [46].

Insulin secretion was glucose concentration‐dependent (Figure 1b) and increased with glucose concentrations up to 10 mM glucose, where the amount of secreted insulin was around three times higher than at 0 mM. The slope of the insulin secretion curve was flatter at lower glucose concentrations (0–4 mM), indicating less active, but not inactive, mechanisms of intracellular insulin secretion below this threshold. At glucose concentrations higher than 10 mM, insulin secretion appeared to level off.

Measuring glucose concentration‐dependent changes in protein abundances upon short‐term exposure poses technical challenges: most proteins are expected to show only small abundance changes, impeding the distinction of true biological responses from measurement noise. To mitigate these challenges, we measured the proteomes of six independently cultured replicates per condition and used a data‐independent acquisition approach to increase the completeness of protein identifications across replicates and conditions. Before cell lysis, all samples were washed twice with the corresponding stimulation media to remove the extracellular proteome, including the secreted insulin. Wash and lysis steps were conducted on‐plate to minimize sample handling time. To ensure analytical precision and to allow normalization based on technical factors, we introduced six process controls (Materials and Methods). Furthermore, to monitor mass spectrometer performance over the run time and to estimate the technical variability of the measurements, we prepared another quality control pool that was injected every 10 samples. The proteomic data were acquired using data‐independent acquisition (DIA) with a 115 min gradient for each sample. Raw data were processed using DIA‐NN [23] using an in silico generated spectral library containing rat and bovine peptides to account for both proteins of interest expressed by the INS‐1 832/13 cells, as well as for potential contaminants present in the medium due to fetal calf serum addition. Only reliably detected precursors were retained for the quantification (Materials and Methods), and bovine proteins were excluded in subsequent analyses.

Three thousand seven hundred and three rat proteins were consistently quantified across the samples (Table S1), with a very low fraction of missing values (0.31%). Measurement accuracy was high, with the median coefficient of variation (CV) among injection quality control samples at 12.71% (Figure 1c). Process quality control samples had higher CVs than injection quality control samples (median at 20.19%), and the distribution of CVs within replicates of the respective stimulation conditions was in between (median at 15.89%) (Figure 1c). Consistency across replicates was similar between all conditions, with median CVs ranging from 13.95% in condition 4 mM to 19.90% in condition 5 mM (Figure 1d). In contrast, the distribution of CVs calculated across all samples was shifted toward higher values (median at 23.88%) compared to the control samples (Figure 1c), indicating that despite the short glucose exposure time, we were able to record biological differences in the beta cell proteomes.

### 3.2 Proteins Show Different Response Patterns to Short‐Term Glucose Stimulation

**FIGURE 2:**
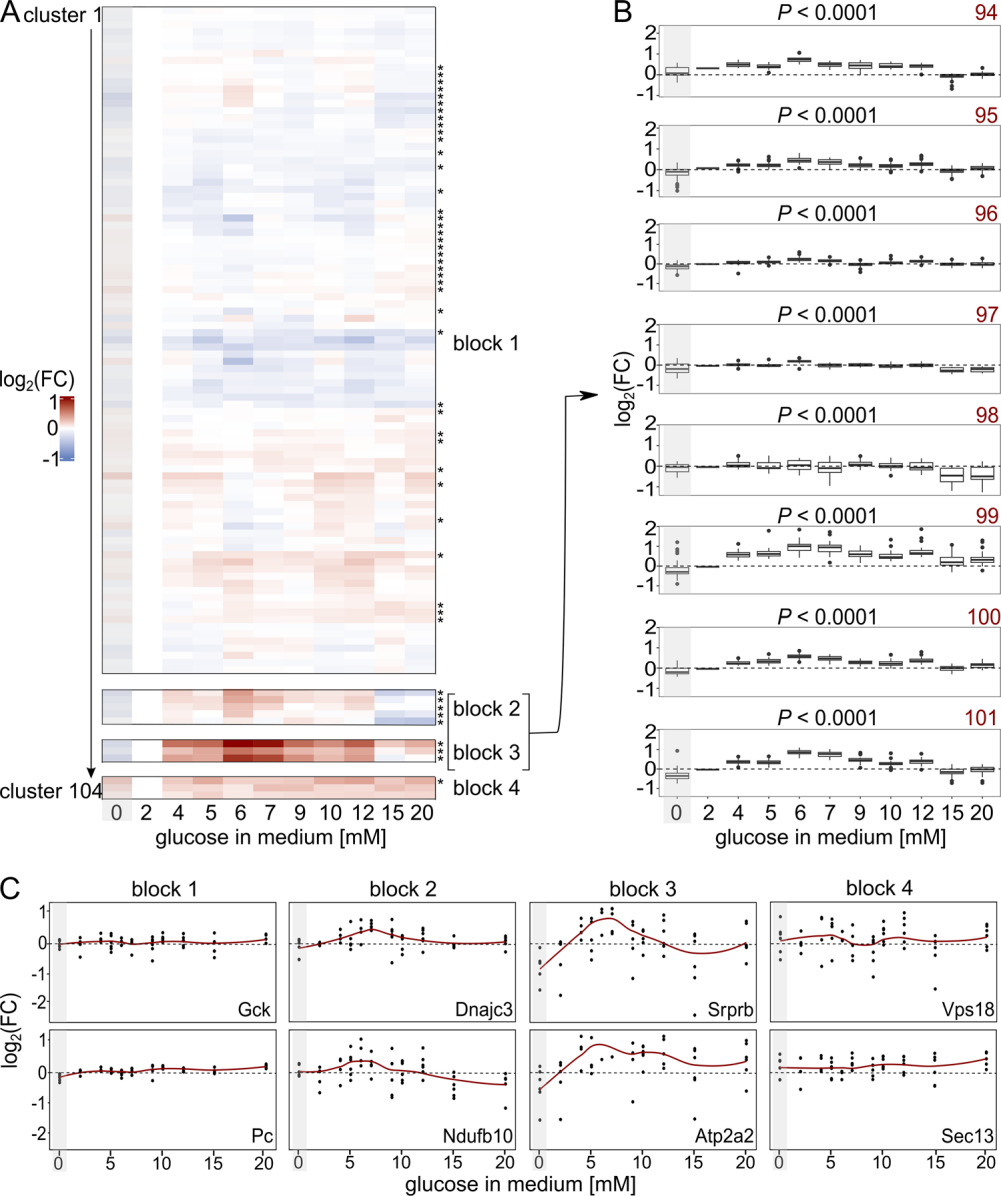
(A) Ensemble cluster analysis of proteomic changes. In total, 104 clusters, and 11 superclusters were grouped into four different blocks. The median of the log_2_ fold changes (log_2_FC) of the proteins in each cluster relative to condition 2 mM is shown. Condition 0 mM is grayed out because it was not included in this analysis. *p* values were calculated by one‐way ANOVA relative to the 2 mM condition, with adj. *p* value < 0.0001 indicated by an asterisk. (B) Variance of clusters in block 2 and block 3, shown as boxplots for each cluster across the conditions. Each facet shows a cluster which is numbered in red on the top right. *p* values were calculated by one‐way ANOVA relative to the 2 mM condition. (C) Abundance patterns of selected proteins (glucokinase (Gck), pyruvate carboxylase (Pc), DnaJ homolog subfamily C member three (Dnajc3), mitochondrial intermembrane space import and assembly protein 40 (Ndufb10), signal recognition particle receptor subunit beta (Srprb), endoplasmic reticulum calcium ATPase 2 (Atp2a2), vacuolar protein sorting‐associated protein 18 homolog (Vps 18), protein SEC13 homolog (Sec13)). Each protein was measured in replicates (black dots). The means of the conditions are shown by the red line.

Next, we identified proteins with similar response profiles across different glucose stimulation conditions. For this, we applied an ensemble clustering approach across all 3703 proteins by combining multiple distance‐based cluster algorithms to account for differences at each time point [25, 47]. The resulting superclusters and clusters of proteins denote similar concentration‐dependent relative proteomic expression profiles (Figure 2a, Table S2). To align better with physiological conditions, we used the 2 mM glucose treatment rather than the zero‐glucose condition as baseline, which was excluded from this analysis. We chose the 2 mM condition as baseline, because the two conditions closest to the 3 mM condition (2 and 4 mM) differed only in the expression of one protein (Figure S1).

The ensemble clustering analysis yielded 104 clusters in total, summarized in 11 superclusters. Eight of these blocks contained proteins that changed only minimally, with median log_2_ fold changes (log_2_FC) not exceeding 0.2 and −0.5 in any condition. These blocks were further summarized into a single block encompassing 93 clusters containing 3207 proteins (block 1, Figure 2a). Thirty‐nine of these clusters showed significant changes (adj. *p* value < 0.0001) of log_2_FC values in one or more conditions relative to condition 2 mM, as tested by one‐way ANOVA. The second block included proteins with a moderate increase in abundance upon glucose stimulation, and a decrease at higher glucose concentrations (block 2, clusters 94–98, 212 proteins, Figure 2a–c). All protein clusters in that block showed a peak at condition 6 mM with a median log_2_FC of 0.1 to 0.4, and reached lower abundance levels with a median log_2_FC of −0.5 at condition 15 mM. The third block contained proteins that followed a similar pattern as block 2 proteins, but with much higher fold changes, peaking at 6 mM with median log_2_FC between 0.6 and 1.0. As in block 2, protein abundances decreased at higher glucose concentrations until returning to baseline expression levels between 0.2 and −0.1 at condition 15 mM (block 3, clusters 99–101, 102 proteins, Figure 2a–c). All clusters of blocks 2 and 3 showed significant changes of protein expression across one or more conditions in a one‐way ANOVA test (adj. *p* value < 0.0001) (Figure 2a,b). The variance within conditions was higher for clusters of block 3 (median interquartile range at 0.47) than for clusters of block 2 (median interquartile range between at 0.31) (Figure 2b).

Block 4 proteins were consistently more abundant (median log_2_FC until 0.4) across all conditions relative to the 2 mM condition, with only one cluster showing significant concentration changes across the conditions (adj. *p* value < 0.0001) (block 4, 182 proteins, Figure 2a,c).

### 3.3 Upregulation of Proteins Implicated in GSIS‐Related Pathways

**FIGURE 3:**
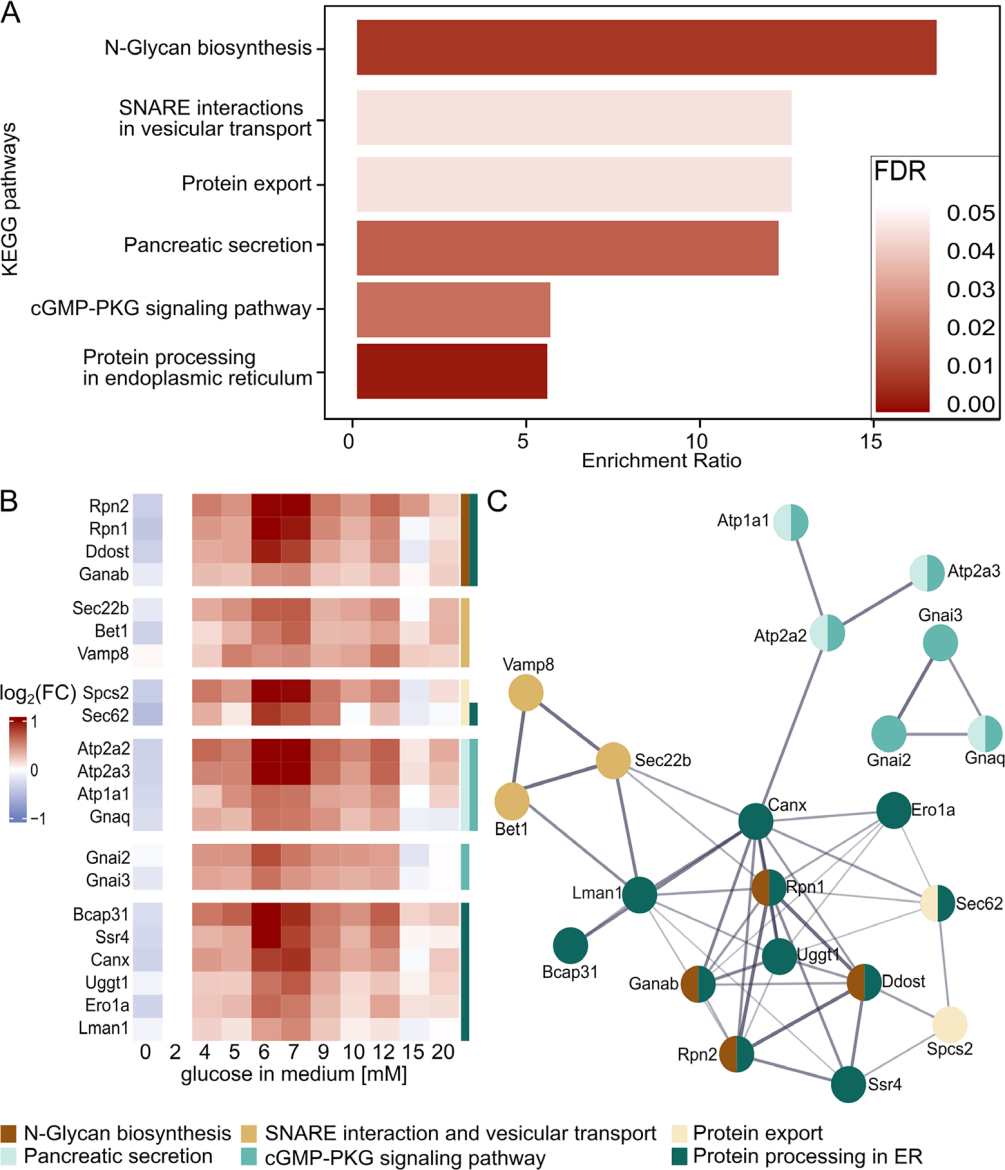
(A) Overrepresentation analysis for KEGG pathway terms for the 102 proteins of block 3. The false discovery rate (FDR) of each enriched pathway is indicated. Only significantly enriched pathways (FDR < 0.05) and pathways associated with pancreatic beta cells are shown (see also Table S4). (B) Heatmap showing the abundance changes of all proteins driving the overrepresentation of pathways of (A) across the conditions. The median of the log_2_ fold changes (log_2_FC) of the proteins in each condition is indicated. Log_2_FCs were calculated relative to protein abundances at condition 2 mM. The color annotation of rows indicates pathways. Proteins are ordered within pathway blocks by descending order of highest maximal log_2_FC. (C) Functional protein association network consisting of all proteins from block 3 that belong to one of the shown overrepresented pathways in (A). Node color indicates the pathways and line thickness data support strength.

To determine whether certain KEGG pathway terms were enriched in different protein blocks, we performed an overrepresentation analysis. All eight sub‐blocks of block 1 were individually run in order to find regulated pathways, but in none of these blocks, any enriched pathways were detected. In combination with the marginal abundance changes, we thus consider all proteins in block 1 as not glucose concentration‐dependently regulated. In contrast, for block 2 we observed an enrichment of several KEGG pathways terms, notably two associated with GSIS in pancreatic beta cells: “Protein processing in endoplasmic reticulum” (enrichment ratio = 4.5, FDR < 0.01) and “Oxidative phosphorylation” (enrichment ratio = 3.1, FDR = 0.05) (Table S3). Similarly, block 3 showed enrichment in multiple KEGG pathways terms (Figure 3a, Table S4). Among the pathway terms with the highest enrichment ratio were several that were expected to play a role in pancreatic beta cells after glucose stimulation, including “SNARE interactions in vesicular transport” and “Protein export” (enrichment ratio = 10.8, FDR = 0.04), as well as “Pancreatic secretion” (enrichment ratio = 10.5, FDR = 0.02) (Figure 3a). The top six enrichments with the enrichment ratio were based on 21 proteins identified in block 3 (Figure 3b,c).

**TABLE 1:** Impact of fatty acid biosynthesis inhibition on GSIS.

Gene of interest	Type of inhibition	Model system	Influence on GSIS	Literature
Acaca	Inhibitor: TOFA	INS‐1 832/13	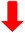	[61]
		Rat pancreatic islets		
	siRNA knock‐down	INS‐1 832/13		[65]
	Beta‐cell‐specific knock out of the *Acaca* gene (βACC1KO)	βACC1KO mice		[66]
Fasn	Inhibitor: Cerulenin	INS‐1 832/13	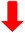	[61]
		Rat pancreatic islets		
	shRNA knock‐down	INS‐1 832/13		[63]
Acsl4	shRNA knock‐down	INS‐1 832/13	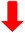	[62]
		Human pancreatic islets		
	siRNA knock‐down	INS‐1 832/13		[64]

The largest number of these proteins (11) belonged to the term “Protein processing in ER”. The interaction network that these proteins (Bcap31, Ssr4, Canx, Uggt1, Ero1a, Lman1, Rpn1, Rpn2, Ddost, Ganab, and Sec62) form was complemented by three other pathways involved in protein processing and transport, which are “N‐Glycan biosynthesis”, “SNARE interaction and vesicular transport”, and “Protein export” (Figure 3b,c). The detected proteins that were part of “N‐Glycan biosynthesis” (Rpn1, Rpn2, Ddost, Ganab) were shared with “Protein processing in ER”, as was one of two proteins from the pathway “Protein export” (Sec62). Proteins from “SNARE interaction and vesicular transport” (Sec22b, Bet1, Vamp8) were uniquely assigned to this pathway, but closely linked to the network around Protein processing in ER (Figure 3b,c). We revealed two more clusters, each containing only three proteins which were all involving the signaling pathways “Pancreatic secretion” and “cGMP‐PKG signaling”. Proteins of “Pancreatic secretion” (Atp2a2, Atp2a3, Atp1a1, Gnaq) were shared with the “cGMP‐PKG signaling pathway”, which further included Gnai2 and Gnai3 (Figure 3b,c). Block 4 did not show any pathway enrichments.

### 3.4 Enzymes of Glycolysis and the Tricarboxylic Acid Cycle Are Not Upregulated During GSIS in INS‐1 832/13 Cells

Interestingly, the block with proteins that showed only minimal abundance changes, block 1, contained many proteins of major metabolic pathways. Important proteins of the glycolysis and the tricarboxylic acid (TCA) cycle with an attributed role in GSIS were found in this block, for example, glucokinase (GCK) and pyruvate kinase (PK). Gck, the enzyme that phosphorylates glucose after entering the cell through the GLUT, showed minimal abundance changes upon glucose stimulation with a maximum log_2_FC of 0.13 at 10 mM glucose (Figure 2c). Parts of the PK protein complex (Pklr and Pkm) were also quantified in our data set, but were not regulated in a glucose concentration‐dependent manner either (Pklr: maximum log_2_FC of 0.02 at 10 mM glucose, Pkm: maximum log_2_FC of 0.01 at 10 mM glucose). Furthermore, pyruvate carboxylase (PC), an enzyme that is critically linked to the TCA cycle as it helps to replenish oxaloacetate, was also not regulated glucose‐dependently (Pc: maximum log_2_FC of 0.15 at 20 mM glucose, Figure 2c). More proteins of these pathways were assigned to block 1, amongst them glucose‐6‐phosphate isomerase, phosphofructokinase, aldolase, triosephosphate isomerase 1, phosphoglycerate kinase 1, phosphoglycerate mutase 1, enolase, citrate synthase, isocitrate dehydrogenase, oxoglutarate dehydrogenase, succinate dehydrogenase, fumarate hydratase, malate dehydrogenase (Table S2). Interestingly, the main glucose uptake transporter in rodent beta cells (GLUT2) was, in contrast to the downstream glycolytic enzymes, sharply upregulated with increasing glucose concentrations (block 3).

### 3.5 Glucose Starvation‐Repressed Proteins

**FIGURE 4:**
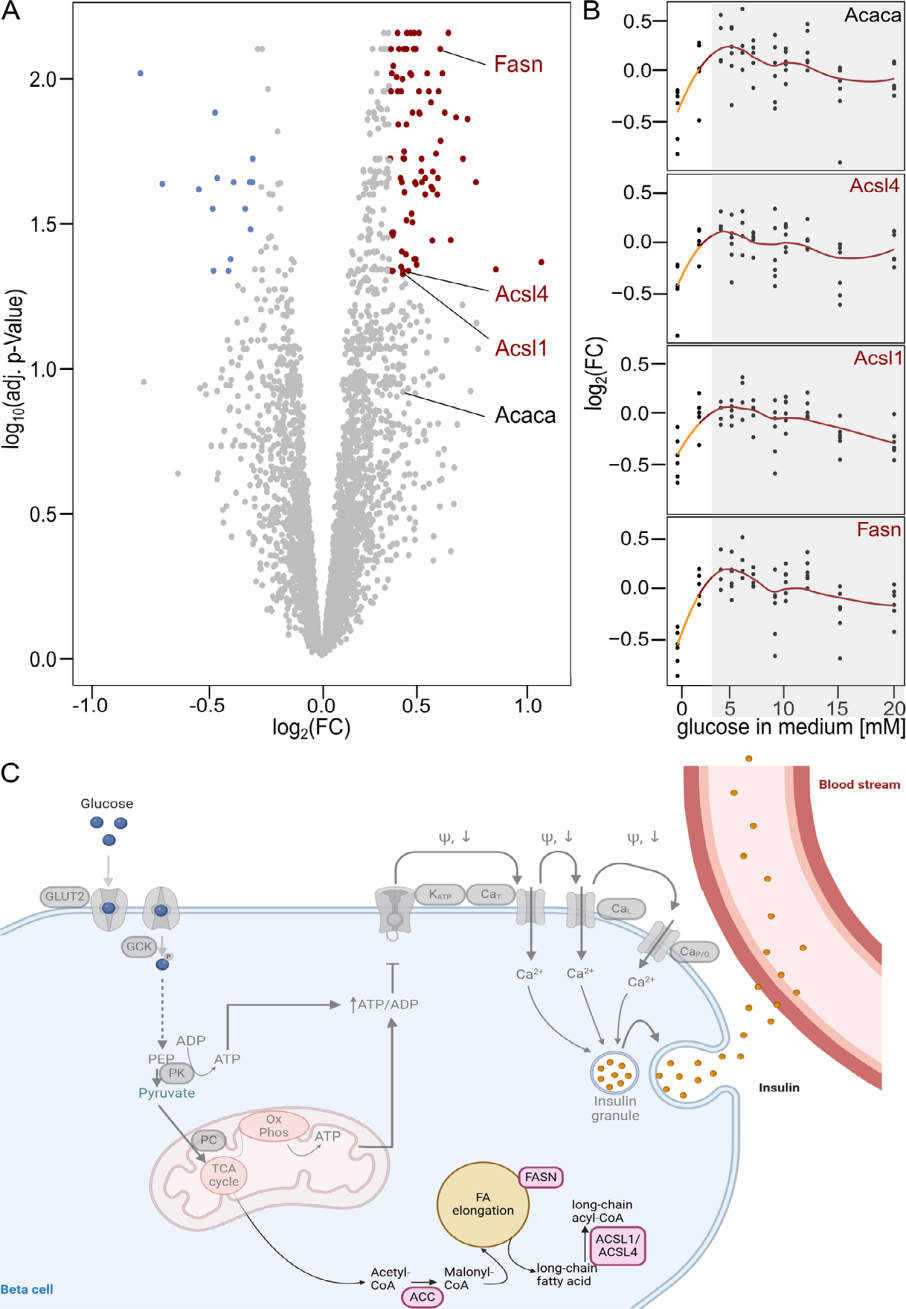
(A) Differentially expressed proteins at 2 mM glucose versus 0 mM glucose (Vulcano plot). All significantly downregulated proteins (log_2_FC < −0.3, adj. *p* value < 0.05) are shown in blue, all significantly upregulated proteins (log_2_FC > 0.3, adj. *p* value < 0.05) are shown in red. *p* values were adjusted for multiple hypothesis testing using the Benjamini–Hochberg correction. The four annotated proteins belong to the fatty acid biosynthesis pathway. (B) Abundance patterns of the four proteins from (A). Each protein was measured in replicates (black) of which the mean was calculated (red line). The orange line highlights the abundance change from 0 to 2 mM. Conditions 4–20 mM are grayed out because they were not included in the differential expression analysis. (C) Proteins (light pink) that were identified as part of the overrepresented KEGG pathway fatty acid biosynthesis (scheme). The canonical GSIS pathway is shown in transparent gray (GLUT2, glucose transporter 2; GCK, glucokinase; PEP, phosphoenolpyruvate; PK, pyruvate kinase; PC, pyruvate carboxylase; TCA cycle, tricarboxylic acid cycle; OxPhos, oxidative phosphorylation; ATP, adenosine triphosphate; ADP, adenosine diphosphate; ACC, acetyl CoA carboxylase; FASN, fatty acid synthase; ACSL, long chain fatty acid CoA ligase; FA, fatty acid; K_ATP_, ATP dependent potassium channel; CA_T_, t‐type calcium channel; CA_L_, l‐type calcium channel, CA_P/Q_, P/Q‐type calcium channel; ω, membrane potential).

We next investigated the proteome response to zero glucose by performing a differential expression analysis between 0 and 2 mM. We identified 85 proteins as significantly upregulated (log_2_FC > 0.3, adj. *p* value < 0.05) and 15 proteins as significantly downregulated (log_2_FC < −0.3, adj. *p* value < 0.05) (Figure 4a, Table S5). An overrepresentation analysis did not reveal any pathway enrichment among the downregulated proteins. Among the upregulated proteins, the KEGG pathway term “Fatty acid biosynthesis” was enriched (enrichment ratio = 23.4, FDR = 0.046). At the individual level, the abundance patterns of proteins assigned to this pathway, fatty acid synthase (FASN), acetyl CoA carboxylase 1 (ACCa, encoded by the Acaca gene), and long‐chain fatty acid CoA ligase 1 and 4 (ACSL1 and ACSL4), showed a strong increase up to 4 mM of glucose (log_2_FC between 0.3 and 0.5 compared to condition 0 mM), followed by slight decrease upon higher glucose concentrations (Figure 4b).

## 4 Discussion

GSIS has previously been intensively investigated at the metabolic [15, 48, 49] and electrochemical level [50, 51, 52]. Here, we characterized the behavior of the beta cell proteome at an early time point of GSIS by treating INS‐1 832/13 cells with varying glucose concentrations from 0 to 20 mM for 30 min. We revealed the upregulation of GSIS‐related pathways during stimulation, and showed that fatty acid biosynthesis proteins are significantly downregulated under zero glucose conditions. In total, using DIA proteomics, we recorded dosage‐dependent abundance profiles of 3703 proteins across eleven conditions, with only 0.31% missing values. Previous data‐dependent proteomic studies that used similar instrumentation and elution gradients to measure proteomes of INS‐1 832/13 beta cells identified around 3066 protein across four samples [53], 5214 proteins across eight samples [54], and 5808 proteins across 136 samples [21]. Whereas these previous studies quantified the proteome to a comparable extent as our study, they did not investigate the early proteome response to glucose stimulation. Notably, at this early sampling point, we observe a relatively high proteome variability within biological replicates, which herein was mitigated by including a high number of replicates. However, we speculate that this biological variability may point to intrinsic variability in GSIS. Despite the early sampling, we detected a significant proteomic response. Although hierarchical regulation is usually expected to be a relatively slow process, protein abundance can also be controlled by faster mechanisms [55, 56]. For example, protein abundance is not only controlled by synthesis, but equally through protein degradation, including degradation via the ubiquitin proteasome system and the autophagic pathways, as well as secretion and translocation.

In our dataset, proteins showing glucose concentration‐dependent abundance changes formed distinct clusters in an ensemble clustering approach. Among the strongly changing proteins, we detected many that are intuitively linked to glucose‐dependent regulation or insulin secretion, as they are associated with “Pancreatic secretion”, “Protein export”, and “SNARE interaction and vesicular transport”. Moderately changing proteins were overrepresented for the KEGG pathway terms “Protein Processing in ER” and “Oxidative phosphorylation”.

Another response pattern resembled a “switch‐on” effect, characterized by a strong increase of abundance from 0 to 2 mM, followed by a slight increase at 4 mM and a slight decrease of abundance at higher glucose concentrations. Only one KEGG pathway term—Fatty acid biosynthesis—was significantly enriched among proteins of this response pattern. The role of fatty acid biosynthesis in GSIS has been discussed in the literature [57, 58, 59], and malonyl‐CoA, the product of ACC, has been suggested to be a regulative metabolic coupling factor [60]. A study focusing on the short‐term metabolic response of pancreatic beta cells to glucose exposure [17] revealed a strong increase in malonyl‐CoA with a simultaneous decrease of acetyl‐CoA. Interestingly, in this previous study, the abundance of malonyl‐CoA is rising gradually with increasing glucose concentrations [17]. The protein expression patterns we detected only partially follow the pattern of the metabolic changes. For example, we observed the expression of ACCa to be switched on at low glucose concentrations but staying consistent over higher glucose concentrations. This observation is consistent with the hypothesis that ACC could act as a coupling factor in beta cell metabolism through one of two mechanisms: either ACC is regulated at a level beyond protein expression, or with upregulated expression of the protein in the presence of low glucose concentrations, the maximum capacity of the enzyme is not reached even at high concentrations. The same trend extended to other proteins participating in fatty acid biosynthesis, such as FASN and ACSL, which exhibited similar abundance patterns as ACCa in our data with an even stronger log_2_FC at 2 mM glucose.

Notably, previous studies highlighted the significance of ACC, FASN, and ACSL4 in GSIS (Table 1). Inhibition of ACC and FASN by 5‐(tetradecyloxy)‐2‐furoic acid (TOFA) and cerulenin, respectively, leads to reduced GSIS in both rat pancreatic islets and INS‐1 832/13 cells [61]. Additionally, several in vitro studies employing knock‐outs in the INS‐1 832/13 cells indicated that GSIS induction depends on the expression of ACC, FASN, or ACSL4 [62, 63, 64, 65]. The results from the Acaca gene knock‐out were further validated in in vivo mice models [66]. The role of ACSL4 was confirmed by knock‐down experiments in human pancreatic islets [62]. In summary, the most induced proteins of our proteomic data set have been associated with GSIS independently across different model organisms, aligning with our hypothesis about their critical role.

The switch‐on pattern may be associated with regulatory mechanisms that exhibit a threshold effect. One such mechanism could operate at the transcriptional level. Several transcription factors regulate GSIS, such as Pancreatic/duodenum homeobox protein 1 (Pdx‐1), Forkhead box protein O1 (FoxO1), Transcription factor MafA, Sterol regulatory element‐binding protein 1 (SREBP‐1c), Carbohydrate‐responsive element‐binding protein (ChREBP), and Peroxisome proliferator‐activated receptors (PPARs). SREBP‐1c, ChREBP, and PPARs regulate enzyme biosynthesis from fatty acid metabolism [67, 68, 69]. Activity of transcription factors themselves is usually controlled by phosphorylation processes rather than increase of protein expression, as phosphorylation is faster. Sacco et al. [14] detected phosphorylation of Pdx‐1, MafA, FoxO1, and ChREBP at high glucose concentrations of 16.7 mM after 15 min of stimulation, but did not investigate phosphorylation at lower glucose concentrations. Future studies could investigate transcription factor phosphorylation upon low glucose stimulation to elucidate potential threshold effects driving the rapid upregulation of fatty acid biosynthesis proteins observed in this study.

Furthermore, we would like to note that many protein pathways prominently involved in the triggering phase of GSIS, such as enzymes of glycolysis and the TCA cycle, did not show any abundance changes. This observation is consistent with a transcriptomic study that found that expression of genes associated with these processes is unaffected by rising extracellular glucose concentrations after 1 h of incubation with MIN6 cells [13]. Thus, while processes like vesicle formation and fatty acid metabolism seem to involve regulatory processes that act via enzyme abundance changes, the central metabolic processes involved in GSIS seem to be regulated mostly via specific changes in activity and localization, at least at early time points and in the studied cell line model.

## 5 Limitations

The INS‐1 832/13 beta cell line is one of the most popular models in GSIS research, but using a cell line as a model comes with inherent limitations [70]. Although INS‐1 832/13 cells are a useful and frequently applied model to study beta cell function, they can, like other cell models, not universally reflect beta cell function as it occurs in vivo. The data presented in this manuscript needs to be interpreted in this way, and will inevitably contain elements which are specific to this cell model. In order to generalize the rapid response of FA metabolism enzymes, it will thus be necessary to compare our results to data that could be collected in a similar fashion in other cell models, primary tissue, and in vivo data. To facilitate such comparisons, we have made all data and analytical procedures openly available (Materials & Methods, Table S1).

Any in vitro experimental design simplifies the physiological complexity of GSIS, not at least because in vivo, GSIS is influenced by a broad range of secretion‐stimulating factors. Although glucose is the primary insulin secretagogue, insulin secretion is also modulated by other hormones, and metabolites like amino acids and lipids [71, 72]. One important player is glutamate, which is known to support insulin secretion strongly in the presence of glucose by inducing insulin granule priming [73, 74]. We control for a potential influence of glutamate during GSIS by maintaining constant levels of 2 mM glutamine, the cellular precursor of glutamate, in our incubation media, including in the zero‐glucose condition (Material & Methods). Although we have excluded glutamate to be responsible for our observations, we cannot conclude that an induction of fatty acid metabolism is specific to glucose stimulation. It would be interesting to further evaluate with more experiments whether the “switch on” effect on fatty acid biosynthesis is specific to glucose uptake, or whether it is a more general mechanism induced by other stimuli or stress responses.

Lastly, it is important to mention that proteomic measurements as conducted give insights into protein abundances, that is, the net of protein biosynthesis and degradation, but not about changes in turnover, localization, and activity. Thus, the abundance changes detected will unlikely translate in a 1:1 change in the total activity of the enzymes quantified.

## 6 Conclusion and Outlook

The ongoing debate about glucose‐regulated beta cell metabolism narrowly focuses on the generation of ATP to open K_ATP_ channels, while overlooking other biological processes that could potentially support GSIS [8, 9, 10]. Our data set, along with others, indicates the involvement of additional pathways immediately following glucose stimulation, such as fatty acid biosynthesis. But how could an increase in fatty acid metabolism support GSIS? We speculate about two possibilities.

First, vesicle‐mediated insulin secretion is dependent on membrane lipids that need to be replenished. Increased fatty acid biosynthesis might be required to support this process. By performing ^14^C tracing experiments in the INS‐1 832/13 cell line, MacDonald et al. showed that strong incorporation of ^14^C into lipids happens within 30 min [61]. Furthermore, the levels of specific lipids, namely, cholesterol ester, phospholipids, and triglycerides were most changed. The authors of the study argue that synthesis of lipids is necessary for the transport of insulin in vesicles, and the modification of lipids in cellular membranes for vesicle fusion [61]. Tracing of ^13^C labelled glucose with subsequent LC‐MS analysis furthermore showed a rapid incorporation of glucose derived carbon into acetyl‐CoA and malonyl‐CoA, as well as into phosphatidic acid and diacylglycerol [17], which are important precursors for phospholipids that are incorporated into the membrane.

Second, increased glucose catabolism during GSIS creates a problem with electron flow, which an increase in fatty acid biosynthesis might counteract when acting as an electron acceptor. Oxidation of FFA influences oxidative stress in the cell, and therefore, measuring reactive oxygen species (ROS) in presence of FFA could give insight into the state of the redox balance of pancreatic beta cells. Superoxide and H_2_O_2_ production have previously been measured under normoxic conditions over a glucose concentration gradient in islets [75], demonstrating that ROS levels rise until 10 mM glucose, but decrease at higher glucose concentrations. Cellular ROS levels could be regulated to prevent toxic accumulation, while small amounts of ROS could play a role in signaling to channel fuel excess into lipid storage [75].

As a future direction, measuring ROS and the composition of lipids under hypoxic conditions could give interesting insights on the role of FFA in GSIS as well. Under hypoxic conditions, oxygen is missing as the final electron acceptor for the electron transport chain (ETC), which leads to a decrease in ETC activity, and to accumulation of NADH and FADH. As beta oxidation needs NAD^+^ and FAD^+^ as cofactors, it stops under hypoxic conditions, creating a state where FFAs are not oxidized. If electron flow persists under hypoxic conditions, it is plausible that FFA production is used to maintain the redox balance. Another way to evaluate the role of FFA in GSIS is by computationally modelling the redox state of pancreatic beta cells. The role of long‐ and short‐term exposure of FFA in presence of glucose on pancreatic beta cells has been modelled, where acute exposure of FFA increased GSIS due to accelerated FFA oxidation and a consequently accelerated phosphoenolpyruvic acid (PEP) cycle [76]. Future experiments could adapt this model to include the role of newly synthesized FFA and their role in early GSIS. We hope our dataset stimulates further studies to investigate these possibilities.

## Conflicts of Interest

M.R. is a founder and shareholder, and M.M. is a consultant and shareholder of Eliptica Ltd. All remaining authors declare no competing interests.

## Supporting information




**Supplementary file 1**: pmic70005‐sup‐0001‐SuppMat.docx.

## Data Availability

Raw and processed mass spectrometry data have been deposited at the ProteomeXchange Consortium via the PRIDE partner repository with the dataset identifier PXD053750. Functions used for clustering analysis are available at https://github.com/OliverLemke/ensemble_clustering.
