# Supplementary figures and images for: An INS‐1 832/13 𝛽‐Cell Proteome Highlights the Rapid Regulation of Fatty Acid Biosynthesis in Glucose‐Stimulated Insulin Secretion
Source: Proteomics. 2025 Jul 20;25(15):13–26. doi: 10.1002/pmic.70005 (PMC12332333; doi:10.1002/pmic.70005)

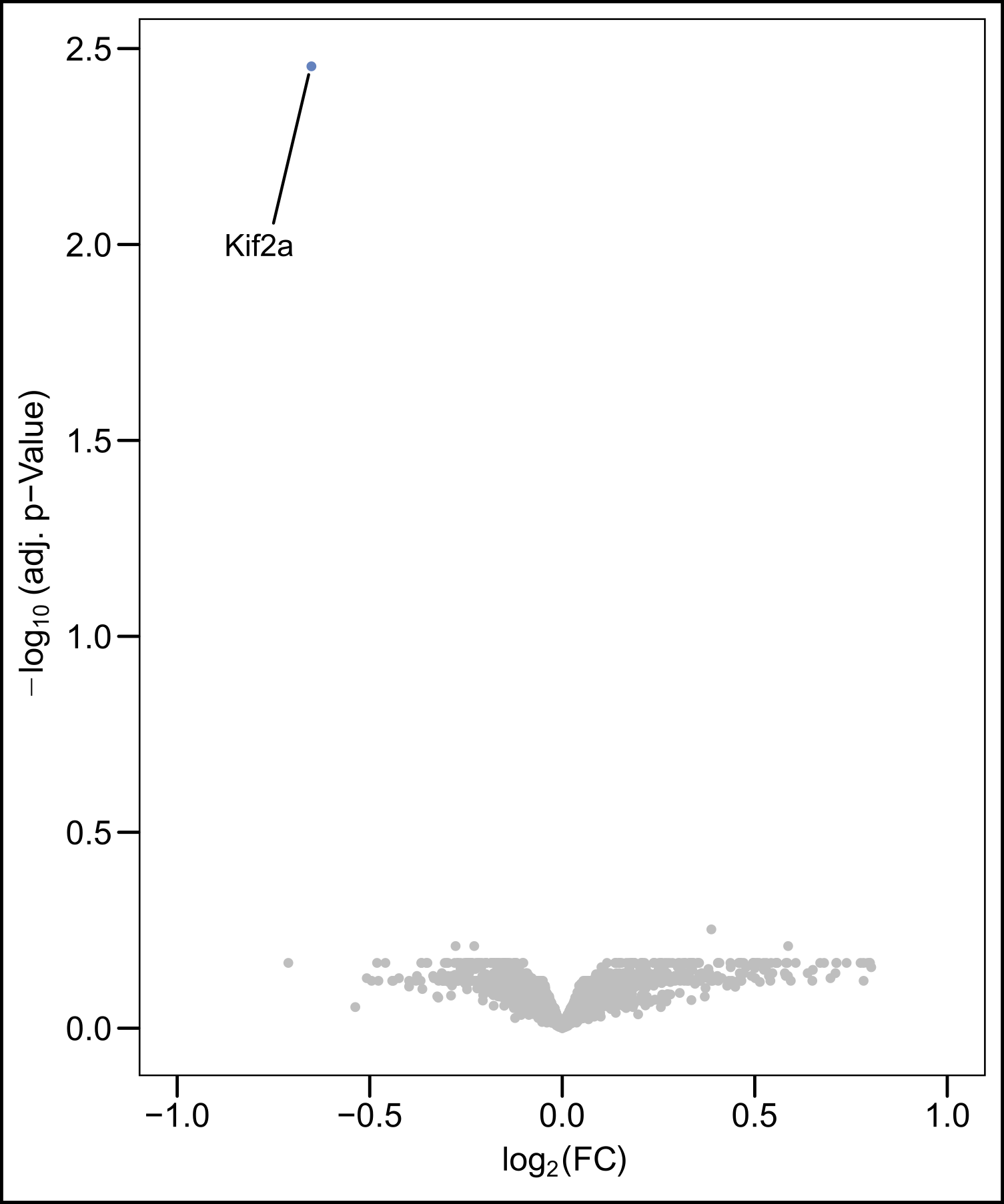

Supplement: Supplementary file 1 — pmic70005‐sup‐0001‐SuppMat.docx. [file PMIC-25--s001.tiff]
